# The Structure of Spatial Networks and Communities in Bicycle Sharing Systems

**DOI:** 10.1371/journal.pone.0074685

**Published:** 2013-09-06

**Authors:** Martin Zaltz Austwick, Oliver O’Brien, Emanuele Strano, Matheus Viana

**Affiliations:** 1 Centre for Advanced Spatial Analysis, University College London, London, United Kingdom; 2 Laboratory of Geographic Information Systems, Ecole Polytechnique Federale de Lausanne, Lausanne, Switzerland; 3 Instituto de Física de São Carlos, University of Sao Paulo, Sao Paulo, Brazil; Universidad de Zarazoga, Spain

## Abstract

Bicycle sharing systems exist in hundreds of cities around the world, with the aim of providing a form of public transport with the associated health and environmental benefits of cycling without the burden of private ownership and maintenance. Five cities have provided research data on the journeys (start and end time and location) taking place in their bicycle sharing system. In this paper, we employ visualization, descriptive statistics and spatial and network analysis tools to explore system usage in these cities, using techniques to investigate features specific to the unique geographies of each, and uncovering similarities between different systems. Journey displacement analysis demonstrates similar journey distances across the cities sampled, and the (out)strength rank curve for the top 50 stands in each city displays a similar scaling law for each. Community detection in the derived network can identify local pockets of use, and spatial network corrections provide the opportunity for insight above and beyond proximity/popularity correlations predicted by simple spatial interaction models.

## Introduction

The role of the Smart City is increasingly seen as being one which incorporates technology, sustainability and quality of life, and the Bike Sharing concept fits neatly under that rubric [1], combining, as it does, low-carbon and low–pollution transportation, sensing technologies, shared societal resources and public health benefits (especially with respect to such key issues as obesity [2]). In this sense the humble bicycle cuts across a number of key issues of the 21^st^ Century City, especially when seen through the Smart Cities lens.

Bicycle sharing offers a low-cost and healthy public transport option for cities across the world, typically allowing users to take special bicycles from automated docking points grouped together as a “stand” or “docking station” in a particular location. The user can then return them to empty docking points at other stands in another location in the same city. There are approximately 450 systems worldwide [3], many of which provide near-real-time data of stand occupation, but only a few of which have released origin-destination (journey) information.

The literature of bicycle sharing systems from around the world takes a number of different approaches to the rich datasets available. [4] explores a subset of the London system’s journey data to analyse spatial “tides” across the city. [5] uses stand occupation data in London to cluster similar stands by temporal behaviour, identifying “railway station-like” and “park-like” nodes in the system; however this work is based on stand occupation data, and does not consider flows. Stand occupation is captured every minute, so at busy times there is a reasonable likelihood that several bikes arrive and several leave, giving only information about the net change of occupation. Previous work by [6] has focused on network analysis and community detection in the Lyon bicycle sharing system, using spatio-temporal characteristics to cluster the network into communities. We would argue, however, that in time-slicing journey data one needs to be extremely cautious about converting *journeys* into *(flow) edges*. These authors circumvent the problem by dealing with flows in terms of *numbers of bikes leaving origin i towards destination j at timeslice k.* This does not represent the number of bikes on a route at a particular time (as these journeys take a finite length of time to complete), but simplifies the process of converting journeys into edge weights.

Network Theory is a branch of empirical science that has evolved from Graph Theory – in short, it examines systems in which *nodes (or vertices)* are connected to one another in some way via *edges.* This breadth of definition has seen it applied to systems as diverse as social networks, co-authorship networks, epidemiological patterns, the internet, links in the world wide web, and many other systems – the review article by Newman [7] and, for technical detail, his textbook [8] are excellent places to start for a interested reader. Early work focused on time-independent, unweighted, undirected (links are reciprocal and not directed), and more recent work has introduced techniques to deal with time-dependence, weighting, direction and spatial factors. Network theory produces a number of results around identifying important nodes and edges, examining the scaling of the importance of these features, and examining any subcommunities within the network. For our study we were interested in turning the flow of bikes into a series of networks; by considering all journeys over the period, we created networks where the nodes were the bike stand locations, and the edges were the flows in each direction between these stands, weighted by the number of journeys carried out on that edge. Work by [9] on shipping and [10] on subway system topologies show ways in which general network representations can be abstracted from the flows and geospatial structures of specific transport networks. The use of network methods to understand spatial systems is a relatively new phenomena –[11]’s work on telecommunications networks established practical measures such as mechanisms for creating spatial null models that real data can be tested against. The motivation behind this spatial scaling is to derive analyses which move beyond Waldo Tobler’s “first law of geography” [12]: “Everything is related to everything else, but near things are more related to each other”. There is the potential for network analyses which neglect spatial embedding to simply highlight proximity effects.

This paper describes a variety of techniques to use network and spatial analysis to understand the flows within bicycle sharing schemes, and compare the activity of five cities. We describe our data sources and preliminary methods to visualise this data, and cover information derived from aggregate data, using a simple spatial model to counter variations in activity density across stands. Creating flow networks allows us to examine network parameters such as strength distribution (the total flows of bikes in, out or through a node), moving on to simple community detection using cluster analysis of outflows. Finally, we describe how basic spatial models can be used to highlight popular routes and spatial community detection can identify networks which are linked more strongly than spatial proximity and stand activity would suggest.

There is a rich range of literature on community detection in networks, for example [13] or [14], and we have applied one of the simpler methods; computationally, a range of approaches are possible because the network is relatively small (no more than 400 nodes). However, these networks do not appear to be sparse in the traditional sense, and this might require different approaches from those that network analysis traditionally takes. Because the networks are based on individual journeys, the raw network is spatial, weighted, directed, time-dependent (in terms of time of day, week and year), and contains self-loops as well as being non-sparse. This means that as a dataset it is amenable to a wide range of analyses by aggregation and simplification. As commented, simplification is necessary in at the very least setting “self-journeys” or “loops” (single-edges journeys starting and ending at the same location) to zero for some analyses, notably spatial networks and community detection.

## Materials and Methods

### Data Sources

Datasets from five cities (London, UK; Boston, MA; Denver, CO; Minneapolis, MN; and Washington, D.C.) were filtered to cover the same range of months (April-October inclusive) in order to sample corresponding seasonal effects. Of course, the climate of each city is distinct and different, but each resides in the northern hemisphere so summer occurs at approximately the same time of year. Some of the schemes (Denver, Minneapolis and Boston) have closures over winter months, so this and data availability limits our reporting period. All data are taken from 2011, with the exception of Boston, which is drawn from 2012 data (which covers March 2012 – September 2012 inclusive) supplemented with October 2011 data.

Table 1 displays summary statistics about the datasets used. In terms of total journeys and stands, London is by far the most active; London also has a smaller average minimum distance between stands, which will tend to influence the proximity statistics below. Each scheme has 213 days of data and over 150,000 journeys.

**Table 1:** Summary statistics for systems expressed as total journeys, illustrating the volumes of data studied.

City	Total [journeys (journeys per day)]	No. of active stands (at end of period)	Journeys on most popular route: [overall (per day)]	Most popular stand: traffic [overall (per day)]	Mean stand traffic per day [total (% of max)]	Max extent [km]	Mean min. sep. [m]
London	3,583,120 (16, 822)	406	3762 (17.7)	64,773 (304)	42 (14%)	10.6	350
Boston	473,240 (2035)	96	2050 (9.6)	20, 092 (94)	21.2 (22.6%)	8.4	360
Denver	168,517 (791)	52	1,149 (5.4)	7649 (35.9)	15.2 (42%)	9.9	620
Minneapolis	213,088 (1000)	116	2520 (11.8)	9,663 (45.4)	35.9 (19%)	16.5	800
Washington D.C.	903,150 (4240)	119	3,061 (14.37)	37, 219 (174.7)	35.9 (20.5)	14.3	700

Per-day metrics (in brackets) are also shown.

### Visualisation

As part of the initial data exploration, we explicitly visualised bike journeys across the cities on particular days. The purpose of this was both to visualise the data as an output in itself, and to begin to identify any strong patterns in the large volumes of source data. Each data point included the origin and destination stand IDs, as well as the start and end times of the journey, and the ID of the bike used by the journey. However, because the bikes themselves do not have location/GPS sensors, precise route information is not available.

To create the theoretical cycle routes between each stand, a software package, *Routino*, was used, with an OpenStreetMap extract of each built-up area, including dedicated cycle infrastructure as well as the road network and path links. Routino uses a configuration profile for each user type. Cyclists will avoid motorways and move on other routes at a constant speed of 20 km/h, except paths and steps where they will move at 5 km/h. Road desirability is factored in, with each road type given a score – trunk roads being given a score of 35%, primary roads 80%, and the smallest roads 95%. Cycleways are given a score of 100% to reflect their attractiveness to bicycle sharing system users. Road routing information, such as one-way streets and turn restrictions, are also applied.

Routino generates a series of latitude/longitude waypoints for the bike’s journeys, allowing multi-stage linear interpolation to create a continuous journey across the city. Individual bikes are drawn as small ellipses at their current position throughout their journeys.

**Figure 1:**
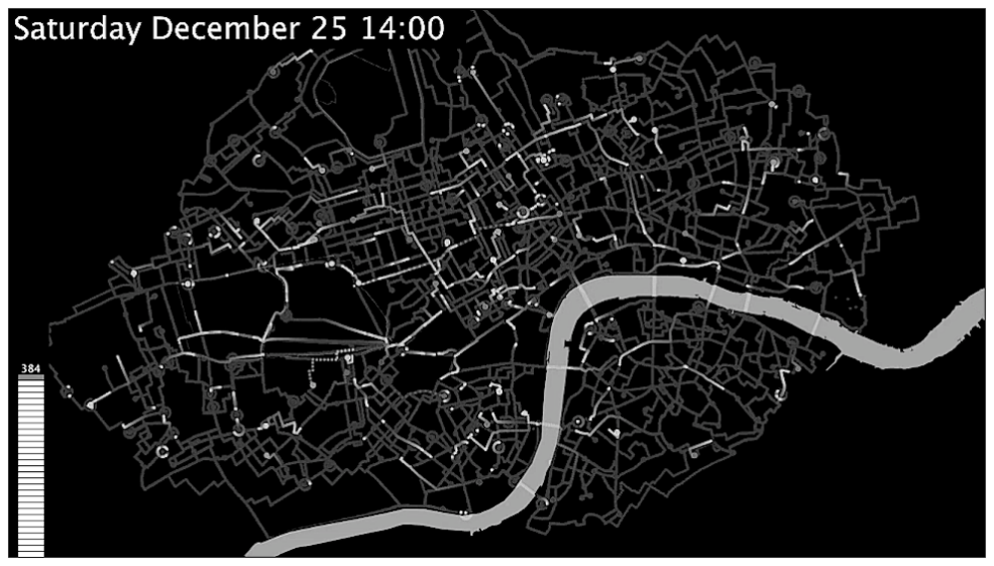
Screenshot from Bike flows animation for December 25^th^ 2010; routing found using Routino based on Open StreetMap Data (see text).

A screenshot from London [Video S1 in Appendix 3 of File S1] is show in Figure 1; similar plots were generated for Boston [Video S2 in Appendix 3 of File S1] and Minneapolis [Video S3 in Appendix 3 of File S1]. Here, a semi-opaque background wipe retains “trails” created by each bicycle, providing a sense of continuity and tracing out the street networks. Self-journeys were represented by traces which orbited the origin/destination stand three times over the journey duration, and then disappeared. These visualisations can be animated for a specific day, or aggregated over multiple days to show a “Monte Carlo-like” picture of the system behaviour e.g. on weekdays [Video S4 in Appendix 3 of File S1] (see also [15] for a complementary visualisation tool).

### Static networks

We generated networks for each system, by counting the number of bikes for each of the source-destination pairs over the reporting period. We further split the data into weekdays and weekends, where we expected different users and patterns due to working (commuter) and leisure users (which we expected to be more dominant over weekends). In each case, this aggregation served to generate a matrix of source-destination flow volumes – we label the origins with the letter *i* and the destinations with *j*; it is notable that this typically contained strong “diagonal” elements (the “self-journeys” mentioned previously, which start and end at the same location). This matrix can be thought of a lookup table of total flows of bikes from origin *i* to destination *j*, and is a mathematical description of a network in the same way that our visualisations provide a spatial or geometrical description of the networks. We will label this object as having N nodes (columns/rows).

**Figure 2:**
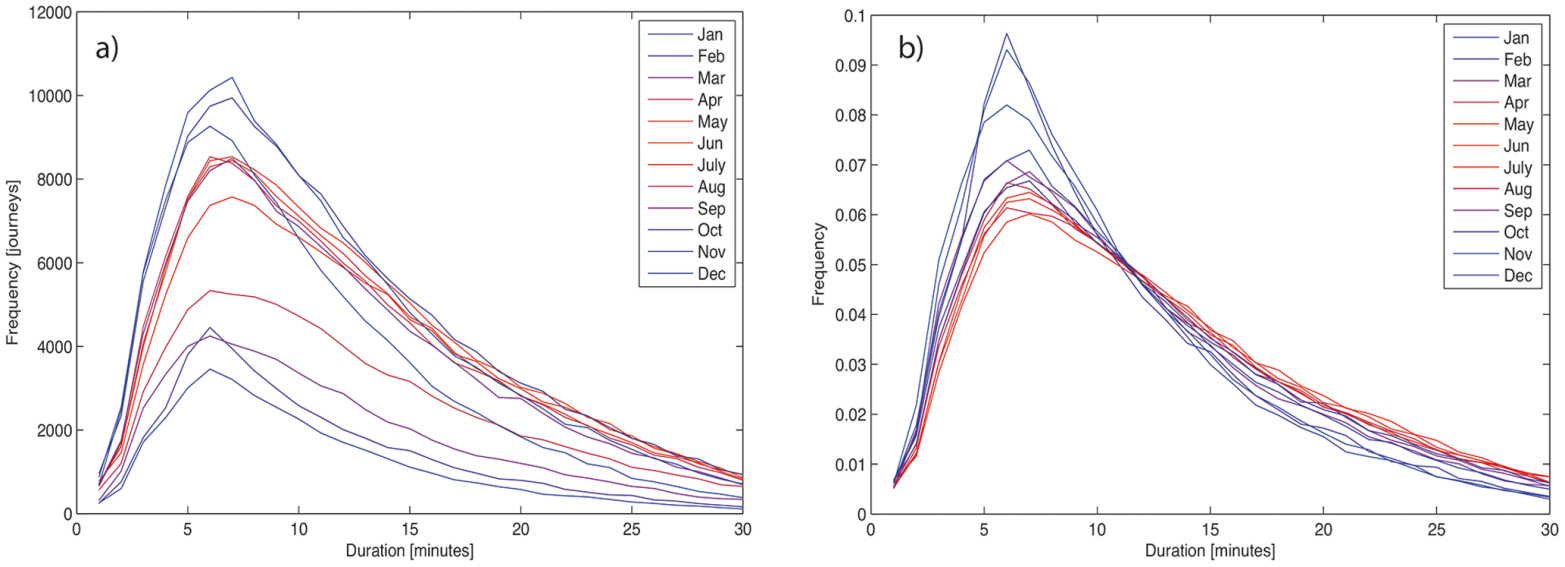
Seasonal dependence of journey duration in Washington, D.C. a) Total journeys by month b) total journeys by month, normalized by area under curve.

These datasets are rich and complex, and can be disaggregated by time of day, season, weekday/weekend, and other factors as well as space. Figure 2 shows, by way of example, the seasonal variations in the duration of journeys in Washington D.C., in terms of both raw journeys (Figure 2a) and data normalized by area under curve (Figure 2b). The winter months show a distribution of slightly shorter journeys, and fewer journeys as a whole. The initial analyses we carry out in this paper sum data over a number of months in order to create an aggregate picture of the network; analyzing individual networks drawn from particular months and/or times of day may illuminate further patterns.

**Figure 3:**
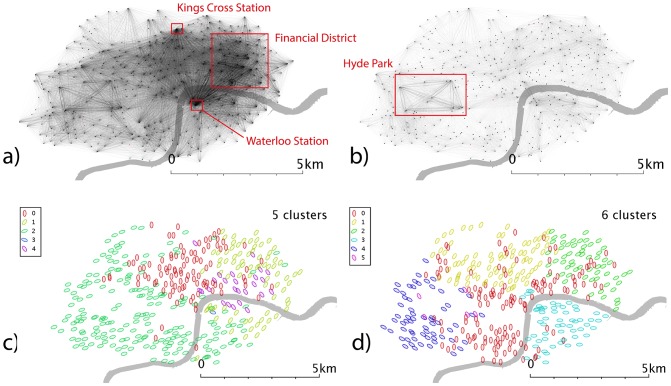
Network and cluster maps for London. Networks are generated from the total number of source-destination flows over the reporting period, split into weekdays and weekends. Community detection is carried out to find sub-networks which are well-linked, as described in the text. a) Weekday network b) weekend network c) community detection of weekday network d) community detection of weekend network. Red dots are stands which show no flows in the reporting period, typically because they were not active.

**Figure 4:**
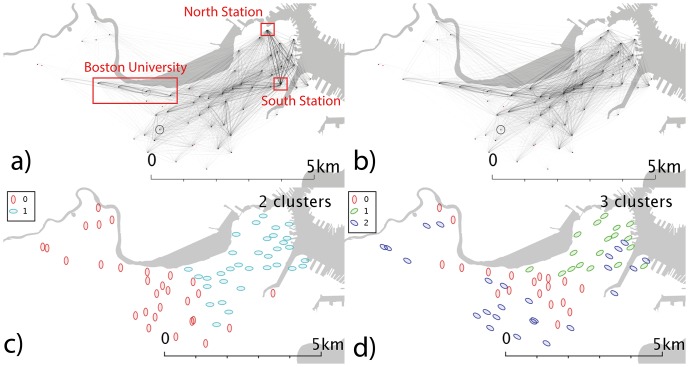
Network and cluster diagrams for Boston, MA. a) Weekday network b) Weekend network c) Communities from weekday network d) Communities from weekend network. Red dots are stands which show no flows in the reporting period, typically because they were not active.

**Figure 5:**
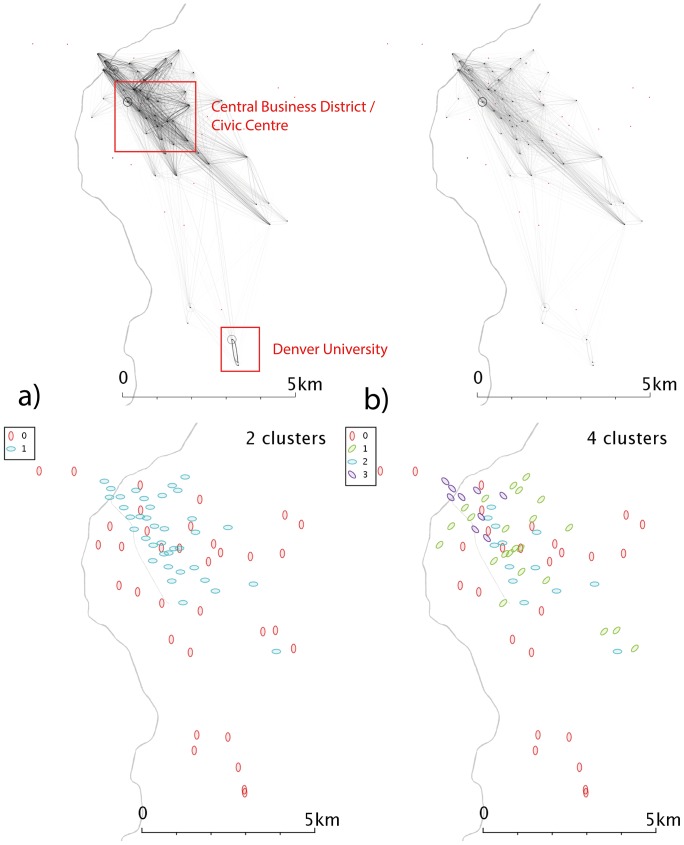
Network and cluster diagrams for Denver, CO. a) Weekday network b) Weekend network c) Communities from weekday network d) Communities from weekend network. Red dots are stands which show no flows in the reporting period, typically because they were not active.

**Figure 6:**
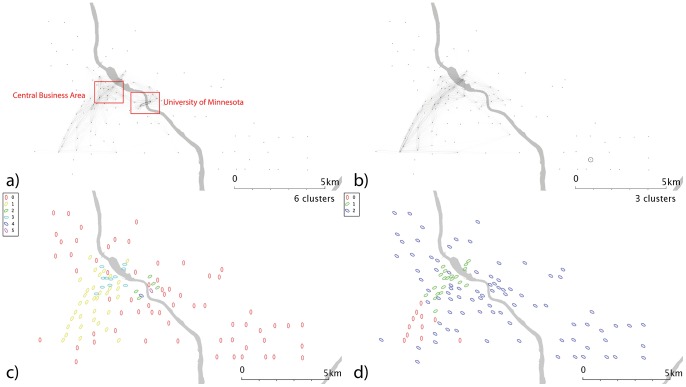
Network and cluster diagrams for Minneapolis, MN. a) Weekday network b) Weekend network c) Communities from weekday network d) Communities from weekend network. Red dots are stands which show no flows in the reporting period, typically because they were not active.

**Figure 7:**
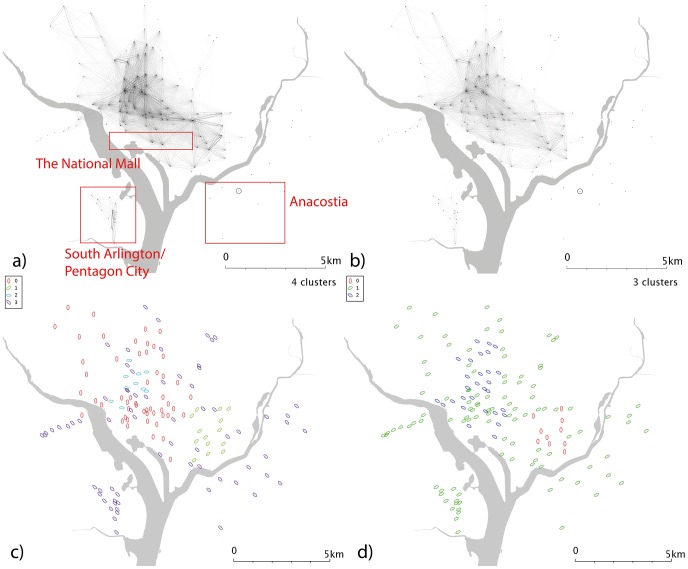
Network and cluster diagrams for Washington, D.C. a) Weekday network b) Weekend network c) Communities from weekday network d) Communities from weekend network. Red dots are stands which show no flows in the reporting period, typically because they were not active.

**Figure 8:**
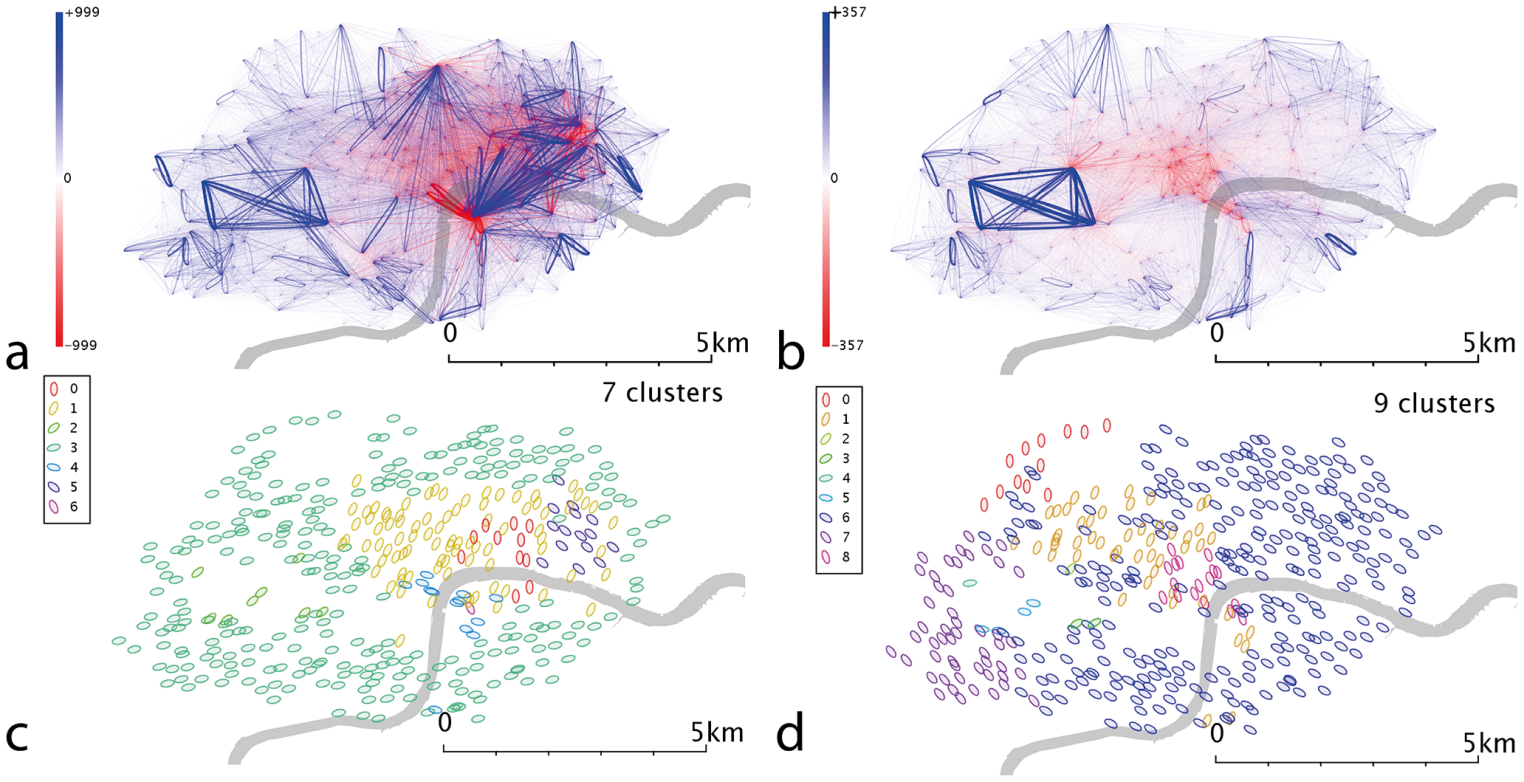
London’s spatially scaled networks and communities. a) Weekday spatial network b) Weekend spatial network c) Communities from weekday spatial network and d) Communities from weekend spatial network. Networks are constructed by subtracting the spatial null model from the raw network data; the above networks demonstrate residuals, i.e. the deviation of the real data from this null model. A blue line represents a flow larger than predicted, a red line represents a flow which is smaller than predicted. The opacity and size of the line represent the degree of deviation from the null model, with the maximum deviation observed in that dataset being shown as completely opaque.

**Figure 9:**
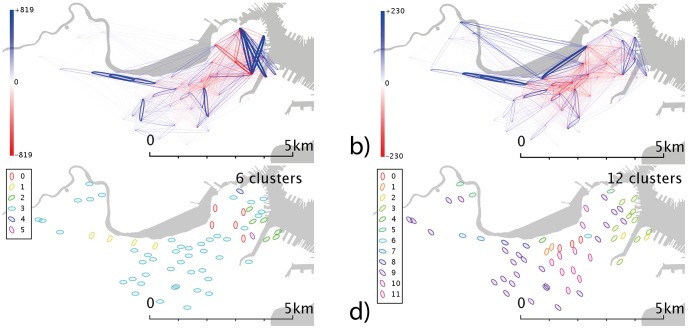
Boston’s spatially scaled networks and communities. a) weekday spatial network b) weekend spatial network c) communities from weekday spatial network and d) communities from weekend spatial network.

**Figure 10:**
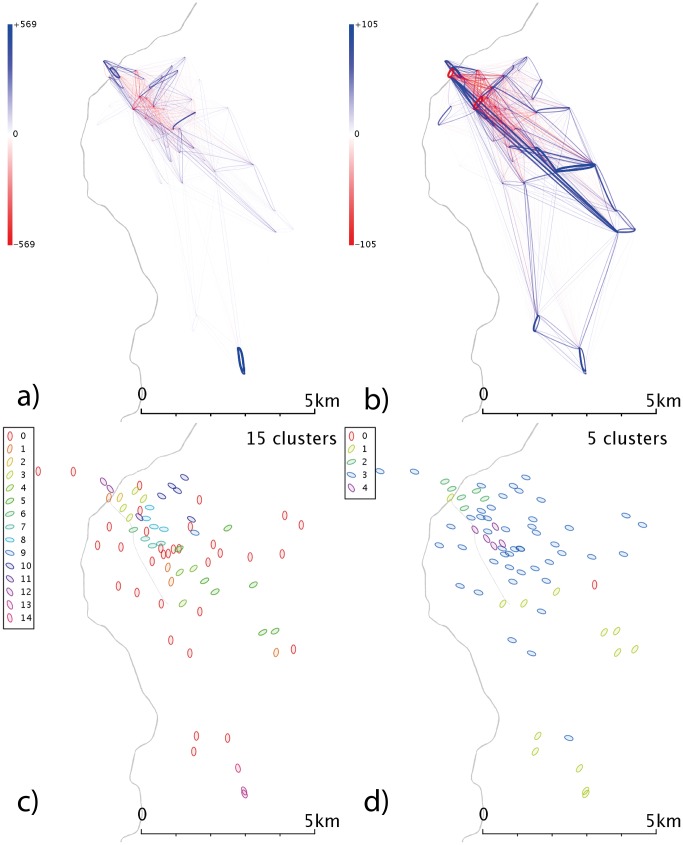
Denvers’ spatially scaled networks and communities. a) weekday spatial network b) weekend spatial network c) communities from weekday spatial network and d) communities from weekend spatial network.

**Figure 11:**
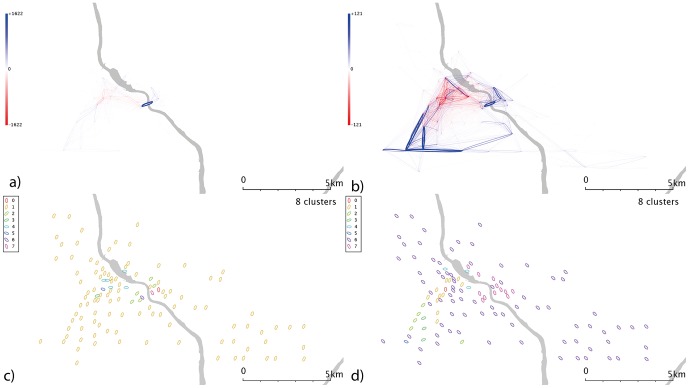
Minneapolis’ spatially scaled networks and communities. a) weekday spatial network b) weekend spatial network c) communities from weekday spatial network and d) communities from weekend spatial network.

**Figure 12:**
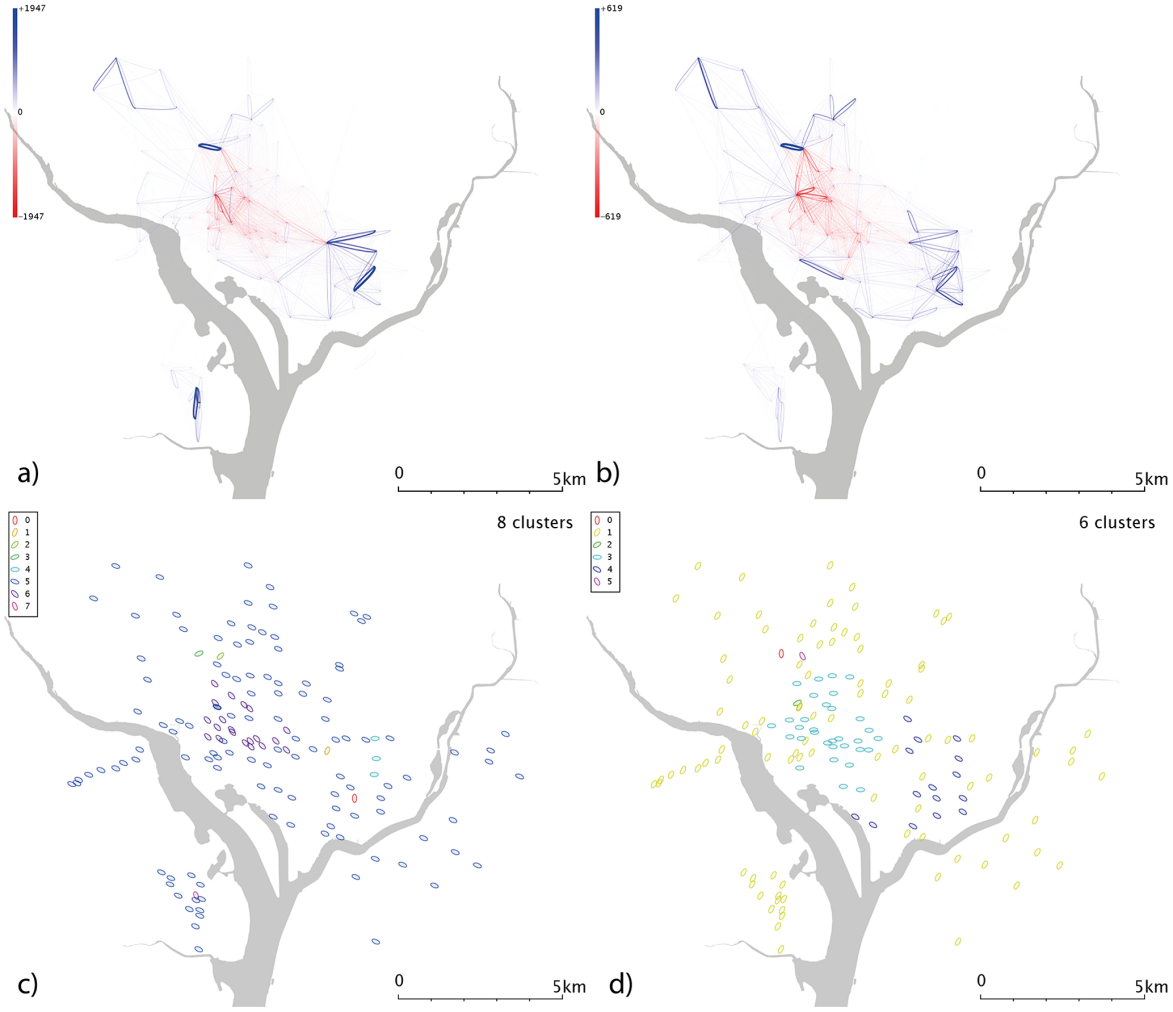
Washington D.C.’s spatially scaled networks and communities. a) weekday spatial network b) weekend spatial network c) communities from weekday spatial network and d) communities from weekend spatial network.

Researchers at London’s City University [16] considered a series of techniques for visualizing these complex systems of flow data, including pseudomatrices, edge bundling, Kernel Density Estimation-type methods and Bezier curves. We adopted a similar method to their Bezier curve techniques, without the explicit size ordering, and using using opacity (alpha - the opposite of “transparency”) as the main weight variable, scaled linearly from 0 to 175 (out of 255) with the edge weight relative to the maximum. This creates a visual grammar where journeys “start” in a straight line and curve into their destination. Representing these very complex datasets across a variety of cities with a variety of edge weight scaling laws is involved, and a detailed discussion is beyond the scope of this paper; all of the static visualizations of network utilize this Bezier curve formalism to denote direction (Subfigures a and b in Figures 3–12) A circle around a stand denotes a self-journey; in some networks, these were the most popular routes.

### Spatial dependence - proximity

The routing generated for the animated visualisations was not used for analysis purposes, as it was judged that it builds in a set of assumptions about route choice, and while reasonable, it infers more information than is available. While a crude measure, Euclidean/Great Circle distance is at least free from these additional assumptions. Self-journeys are problematic in this formulation, as they have zero net displacement but indeterminate journey length. This is not ameliorated by applying routing mechanisms, when there are an astronomical number of redundant paths for a closed loop.

**Figure 13:**
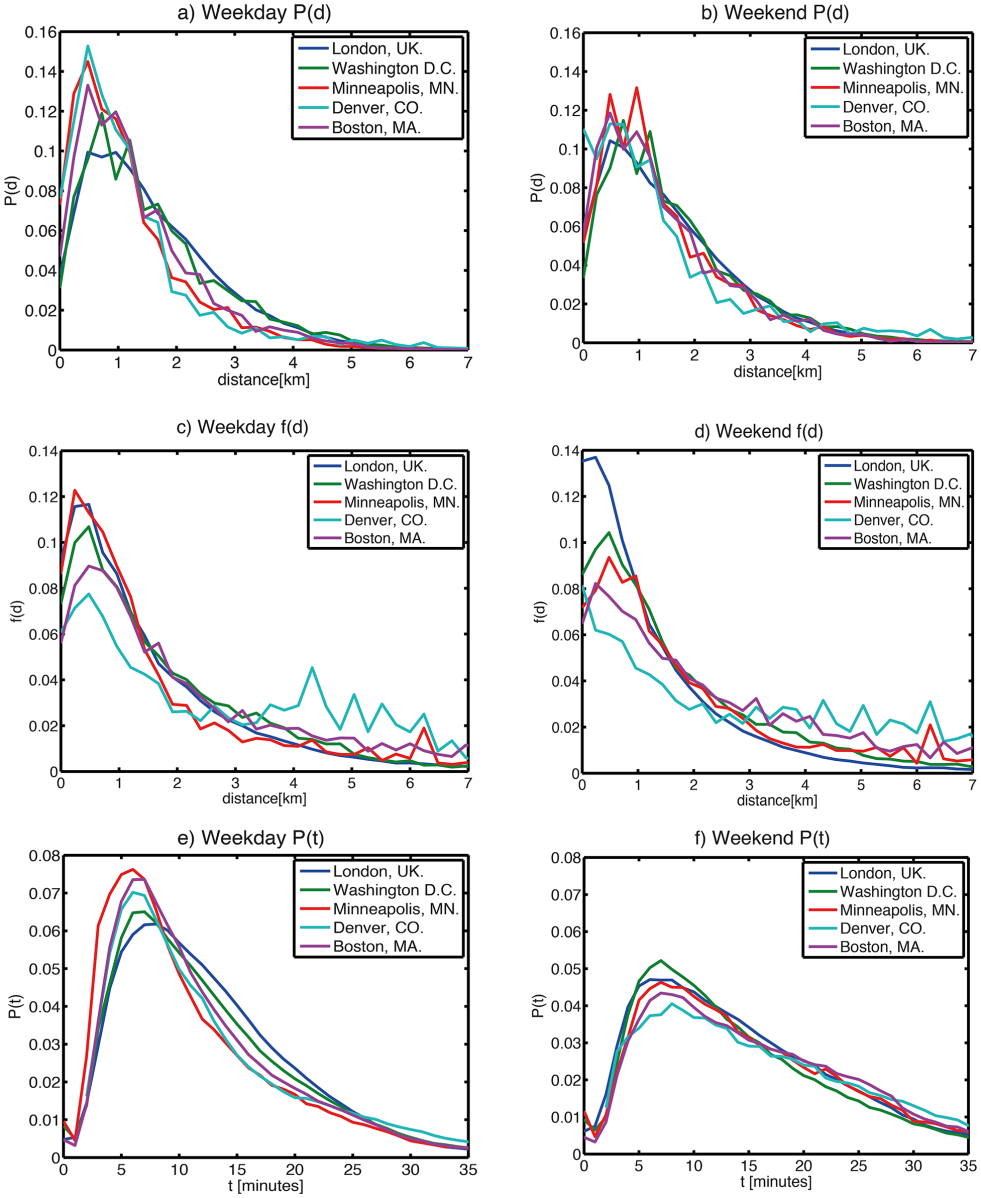
Histogram of journeys in each city. Probability mass function of journeys as a function of origin-destination separation d for a) weekdays and b) weekends; distance decay function of journeys as a function of origin-destination separation f(d), scaled to account for variations in the proximities of popular source/destination stands (see text) for c) weekdays and d) weekends; and Probability mass function of journeys as a function of journey duration for e) weekdays and f) weekends.

We examined the typical journey distances for the cities in question. Aggregating the journey frequency by distance travelled and then dividing by the total number of journeys yields an initial estimate of journey frequency as a function of proximity (weekday and weekend data are shown in Figures 13a and 13b respectively). However, bike stands are not homogeneously distributed through space, and as such the above distribution function might be skewed by a route which moves between a source with a lot of bikes leaving and a destination with a lot of bikes arriving. We might expect that, given a maximum entropy allocation of journeys (i.e. one that assumes as little prior knowledge as possible), the route between these two locations would be well-used, and that this would lead to a commensurate over-representation of this separation in Figure 13(a-b). If instead, we wish to probe an individual’s propensity to travel a location of certain proximity (assuming a constant distance decay function which to first order is independent of absolute start/end location or time), we need to decompose the probability. We can define a spatial (proximity) model of the flows Y^(s)^ such that

1) 
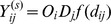



where Y_ij_^(s)^ is the predicted weight (i.e. number of journeys) between origin i and destination j, and f(d) is some displacement-decay function representing people’s preference to travel to some destination displacement d away (note that f(d) is not a probability – if we take Y_ij_^(s)^ to be in units of “bikes travelling on a route”, f(d) will take 1/bikes as its unit). O_i_ and D_j_ are defined by

2) 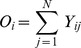



and

3) 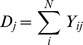



which in spatial interaction modeling can be referred to as the marginal sums, or in network theory, the *out-* and *in-strength* of the node respectively. This is analogous to a naive “gravity” model, and a more sophisticated approach to this problem would be to construct a maximum-entropy model and introduce additional balancing factors into (1). However, given an unknown form of the spatial function f(d), Figure 13(a-b) suggests that it may have a more complex form than an exponential or power-law decay. We instead took an approach favoured by[11], constructing and empirical distance-decay function f(d):

4) 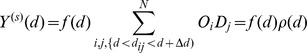



Where Y^(s)^(d) is the aggregated modeled flows for all journeys associated with displacement between d and d+ **Δ**d (where **Δ**d is the chosen bin size). Rearranging (4) yields the distance decay function f(d), shown in 13(c-d) (for weekdays and weekends respectively), normalized by area under curve in order to compare different cities.

### Community detection

Community detection tools allow the identification of network subregions within the bikeshare flow networks which are linked to one another more strongly than nodes from other subregions. One of the simplest methods in terms of implementation is the cluster-algorithm method [14], whereby the flow matrix is treated as a series of column (or row) vectors; then two nodes are judged as belonging to the same community if their links to other nodes are similar (or more simply, if they both have links to the same nodes), in this case based on a Euclidean similarity measure. Agglomerative hierarchical clustering methods allow choices of splits, so it is important to apply a quality measure to assess how meaningful these clusters are. For a directed weighted network described by Y_ij_, we can use the following, usually called the *modularity* after [17] and [18]:

5) 
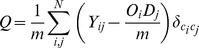



where m is the sum of all edge weights in the network (the time-aggregated version of equation (2)) and the Kronecker delta acts on the community ID for each node {c_i_} (following [19]). The O_i_D_j_/m term represents the aspatial (directed) null model, the hypothesis that edges will be formed in proportion both to the outstrength {O_i_} of the source node and the instrength {D_j_} of the destination node. In a graph in which no communities exist in fact, the number of links within a community that we identify would be exactly the same as the global characteristics of the network; then the modularity for each community would be close to zero, and so would the total modularity. For communities with a large number of links within them and few to other groups, there are preferential factors to link within that community that go beyond linking between “popular” nodes; so Y_ij_ will not resemble the null model, and modularity will tend to be large and positive.

We used this modularity to select an appropriate number of clusters for each city, as it provides a measure of how meaningful these communities identified are, independently of the methods used to detect the communities. As discussed previously, self-journeys were set to 0, to avoid numerous single-stand clusters appearing as a result of such journeys. A Wards hierarchical clustering algorithm was implemented using standard Matlab libraries (linkage and cluster methods) applied to a matrix defined via a call to the MySql database (Table 3).

**Table 3:** Structure of MySQL database.

Table name	Purpose	Key	Other attributes
bikeflow_*cityID*	Record for each distinct journey	Journey ID	Origin & destination stand IDs, start & end times
bikeflow_*cityID­_*namelocation	Information on stand geographies	Stand_ID	Latitude/longitude, descriptive name
bikeflow_*cityID­_*routing	Description of route through network between any pair of stands	Origin & destination stand IDs	Sequence of latitude/longitude pairs describing route between nodes

### Spatial Null Model

Expert et al [11] extended this model to account for the likelihood that, for nodes embedded in physical/geographical space, proximity as well as in/outstrength will dictate the probability of linkages being formed, and an interesting research question is what linkages form above and beyond this spatial/volume dependence. They use a spatial null model to find clusters -equivalently, we can form a network scaled by spatial considerations, so that

6) 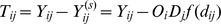



which we expect to be >0 if two nodes are linked more strongly than pure proximity/strengths would dictate. This is represented graphically in Figures 8–12 (subfigures a-b). Here, blue indicates a positive value and red a negative value, and the intensity and weight the numerical value. This is a visual representation related of the method of residues, which has been applied to urban modeling for over thirty years - [20] provides an overview– and creates a link to more recent spatial network theory.

It is possible to form clusters on the basis of this scaled network, using the same cluster analysis as before. This rescaled edge weight allows for rapid calculation of modularity:

7) 
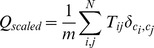



which we can again use to choose the optimum number of clusters/communities. Results are shown in Figures 8–12 (subfigures c-d). Again, self-journeys are set to 0 because distance displacements are not reasonably calculable for these journeys.

## Results

### Time dependence of networks

**Figure 14:**
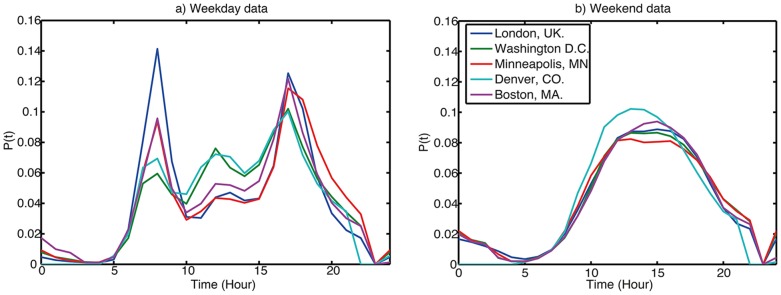
Level of activity (total number of bicycles leaving docks) in the system as a function of hour of day over the reporting period. a) Weekday data b) Weekend data.

Significant variations exist between flow volumes over the course of a day; all cities considered exhibit this characteristic trimodal distribution on weekdays (Figure 14a), with strong peaks at the morning and evening “rush hour” commuting periods, and lunch times, contrasting with the more unimodal peak that dominates weekends (Figure 14b) and we infer to be the hallmark of tourist and/or leisure activities.

We can define *departure* vectors based on the number of bicycles leaving each stand in each hour-of-day period and their destinations, creating an hour-on-hour network. In order to determine the distribution of edge weights and outstrength (here defined as the total number of bikes leaving a node at within some time period) as a function of time, we can use entropy, in its standard definition. Entropy is a measure derived from statistical physics, and latterly from information theory, which can be understood as the level of equality of distribution in a system or dataset; a high entropy corresponds to a state where all entities have similar values, a low entropy where this distribution of values is unequal. In our system, the entities are edges and the values are weights (or nodes and outstrengths). If all edges had equal weight, the entropy tends to a maximum value of ln(1/n); if all edge weights but one were zero, the entropy is a minimum at 0.

If at time t the edge weight distribution is {Y_ij_(t)}, where i is an integer labeling the origin, and j an integer labeling the destination:

8) 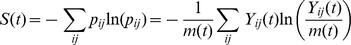



where

9) 
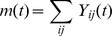



Notably, these entropy measures (not shown) do not vary by more than +/– 10% throughout the course of the day, so the equality of distribution of bikes on routes as a proportion of total bikes being used at that time *does not vary by a large margin over a typical day.* Note that the details of which particular routes are popular *may* change over time; the entropy is a systemic measure which does not relate to individual edges. The variation of the proportional distribution of the bikes to origin stands varies even less (closer to 4% for London). Similar calculations for *stand occupation* (not derivable from these datasets) yield large variations in entropy as commuters redistribute bikes from one highly ordered state to another at peak times (in London, this tidal effect is very clear from stand occupation data - see for example [21]).

### Spatial Patterns

London (Figure 3) shows marked concentrations around the major commuter stations Kings Cross and Waterloo in the weekday data (Figure 3a), as these stations “feed” the financial district in the east (and the centre/”west end”) in the case of Kings Cross. There are marked differences between weekdays (Figure 3a) and weekends (Figure 3b), primarily via a de-emphasis of the financial district at weekends, and a relative increase in traffic in west London and the Hyde Park area (the diamond-shaped flow network to the left of the visualization).

In Boston (Figure 4), the link between North and South Stations in the east of the city is one of the strongest features in the weekday data (Figure 4a); in the west of the city (directly south of the river) there is a fairly strong east-west flow (along Commonwealth Avenue, a station with a high concentration of both Boston University locations and metro stations). At weekends (Figure 4b), the flows to and from North and South station become less dominant, consistent with this relating to commuter behaviours.

Denver (Figure 5) shows very uneven clusters of stands, and the weak flows between the downtown areas to the northwest of the map and the areas to the east and southeast could be linked to the large geographical separations. The flows around the University of Denver campus in the southeast of the city are much more important in the weekday data (Figure 5a) than the weekend (Figure 5b), and at the weekend self-journeys relating to one or two stands are the most popular journeys, suppressing the other routes in the visualisation.

Minneapolis’s weekday flows (Figure 6a) are dominated by movements between two stands named “Social Sciences” and “Kolthoff Hall” - further investigation reveals that this flow occurs across a bridge connecting the two halves of the University of Minnesota Twin Cities (UMN) campus, and so this weekday flow could be largely accounted for by students travelled between lectures and classes. The main apparent difference over the weekend (Figure 6b) is that this flow all but disappears, and so could be related to teaching activities; but a self-journey in the southeast of the city dominates.

Washington DC (Figure 7) shows less dramatic usage changes at the weekend (Figure 7b); during both weekdays and weekends, Washington D.C. itself (which lies in the north part of the network, between the two rivers) is separated from the two more geographically distant cluster in Arlington (south and slightly west on the map) and that the stands in Anacostia (southeast from Washington D.C. and over the river) are much less used. South Arlington/Pentagon City is less well used at the weekend (Figure 7b) than during the week (Figure 7a), consistent with a predominately commuter-led usage.

The systems show similarity in the distribution of journey displacements and durations (13), despite differing climates and spatial extents; Table 2 summarises median travel durations and stand proximities. In the weekday data, Denver shows a much larger median travel distance, despite not being one of the schemes with a larger spatial extent. All of the schemes exhibit a larger median proximity at the weekend apart from London and Washington D.C., even though all schemes show an increase in median journey duration. Notably, this duration change is smallest for Washington D.C. and London, perhaps suggesting that weekday and weekend behaviours are less similar than in other cities. For example, the presence of the Hyde Park cluster in weekday and weekend London data points (Figure 3) reinforces the possibility of leisure and tourism users in both weekday and weekend periods. For each city, the time-behaviour shows consistency with the proximity function, in that there is a nonzero mode; it makes little sense to cycle for a very short distance (much less than 1km), so the function rises to a maximum before decreasing for larger distances/times.

**Table 2:** Median travel times and distances for each city.

City	Median journey duration (weekday) [minutes]	Median journey duration (weekend) [minutes]	Median journey proximity (weekday) [km]	Median journey proximity (weekend) [km]
London	11	14	0.96	0.96
Boston	10	16	1.44	1.68
Denver	11	17	2.16	2.64
Minneapolis	9	15	0.96	1.44
Washington D.C.	11	13	1.20	1.2

### Network Properties

**Figure 15:**
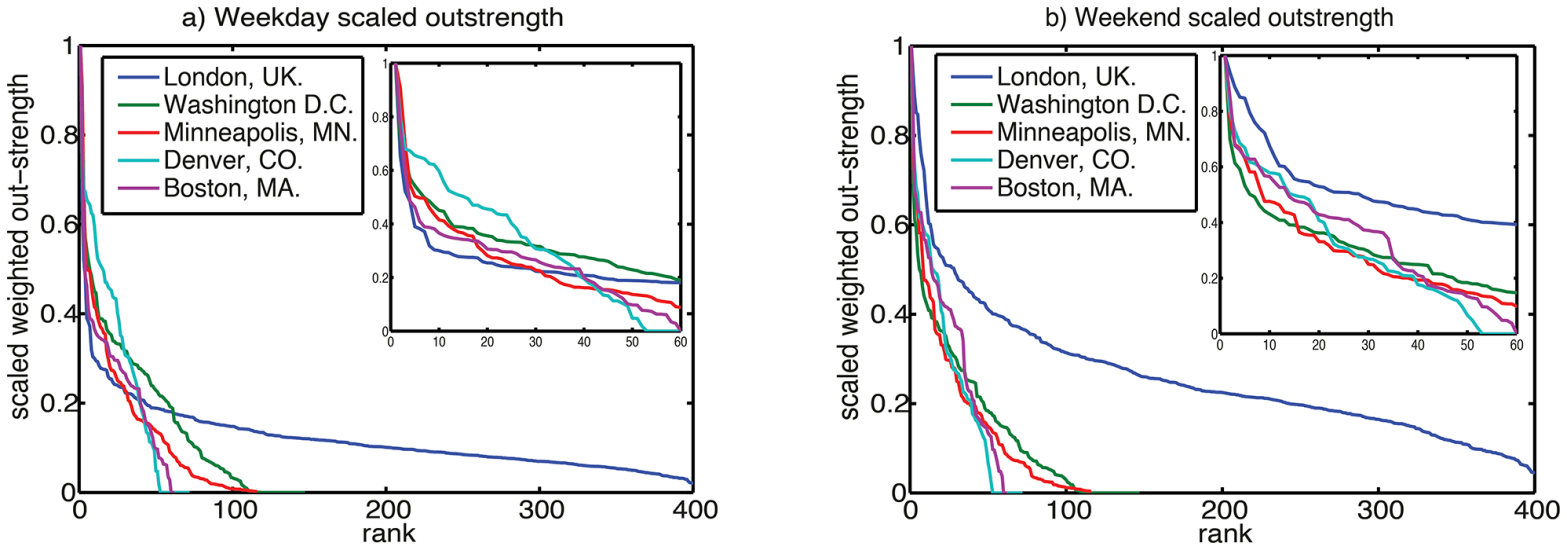
Rank/value plot of stand outdegree for each city. Outdegree is scaled in each city so maximum outdegree is unity. a) All data b) Top 50 stands in each scheme.

**Figure 16:**
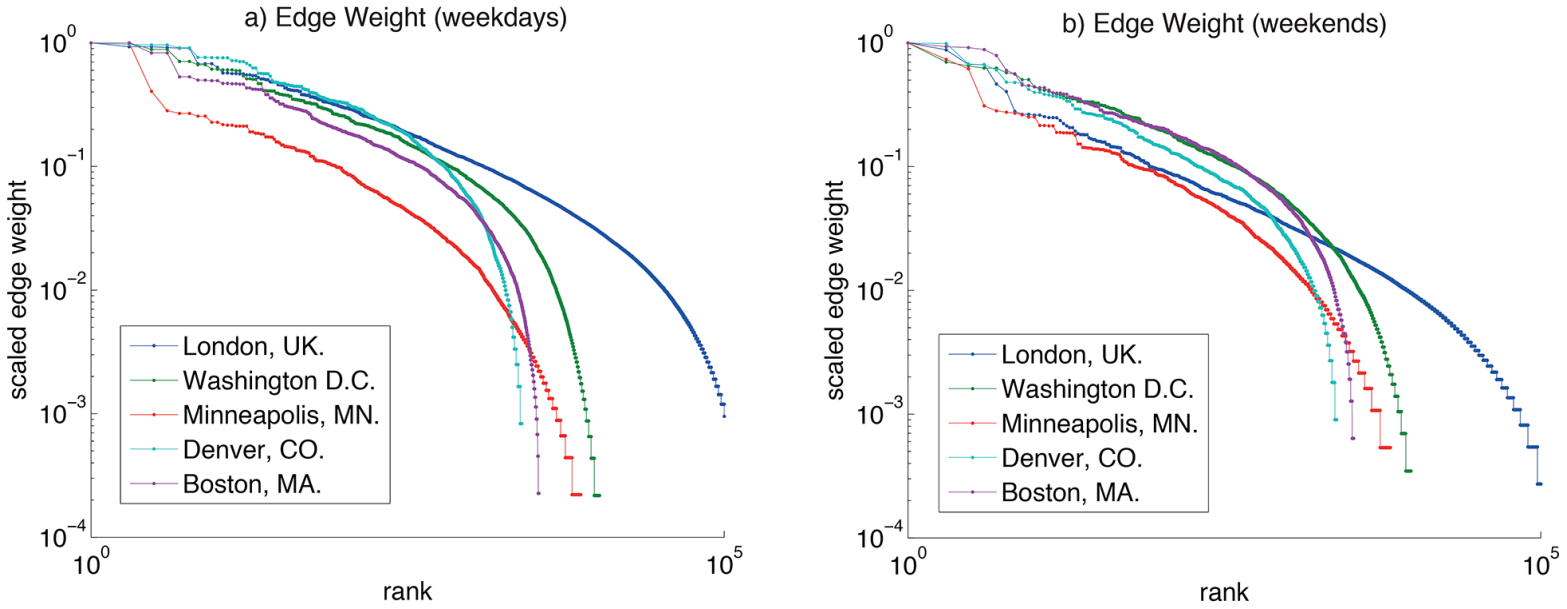
Rank/value plot of edge weight distribution, normalized so maximum edge for each city is unity.

Figure 15 shows the outstrength distribution {O_i_} of the five systems; the data is divided by the maximum outstrength of each scheme, in order to compare them directly. Instrength or combined strength (O_i_+D_i_) could equally well be displayed, as over these aggregation time periods O_i_∼D_i_ (although this is obviously not the case instantaneously). This figure displays a rank/value distribution [22] (which can also be related to the cumulative frequency distribution). Because London’s system has many more stands than the other cities, it exhibits a marked long tail. What is more surprising is the apparent similarity of outstrengths in the top 50 stands in the weekday data (inset, Figure 15a). London, Minneapolis, Boston and Washington D.C. all exhibit similar rank/outstrength distributions in their most used stands. At weekends (inset, Figure 15b), the schemes also appear similar, with the possible exception of London, which has a “flatter” distribution than the other cities. A power law fit does not seem plausible for these datasets – each has a complex structure with linear and nonlinear regions. Nor does it convincingly resemble a log-normal distribution. Ranked edge weight is shown in Figure 16 – these curves do not conform readily to a power law or log-normal behaviour, although the curves imply a quadratic dependence of log-edge weight on log-rank.

### Community detection

Communities are shown in subfigures c-d of Figures 3–7. In the London weekday data (Figure 3c), one can broadly identify groups corresponding to central-east London (where financial services are traditionally based), central-west (more associated with retail), east and west. These seems to cross the river, whereas the weekend data (Figure 3d) exhibits clusters which appear to form more distinct geographical regions, and which are more divided by the river.

In Boston, the weekday data (Figure 4c) shows a distinct east-west split, possibly the eastern region corresponds to commuters to the civic and financial district that lies in the northeast corner of Boston, but a time-analysis would be needed to elucidate that behaviour. Weekend clusters (Figure 4d) are more complex, with a central-south grouping as well as a community in the northeast. For Denver (Figure 5c-d), the clusters shown demonstrate geographical overlap; the weekend data fragments into 4 clusters with no distinct geographical character.

Minneapolis’ weekday data (Figure 6c) show a series of clusters, including one corresponding to the university campus area; a cluster of similar size to the northwest; and a larger group which covers most of the city but lies predominantly to the east. The larger group to the southwest of the centre may be linked the “the wedge”, a region of Minneapolis favoured by young professionals. If so, this cluster could highlight commuters from “the wedge” to downtown, and its associated businesses and entertainments. Weekend data (Figure 6d) splits the city into 3; a large, generic cluster; a small southwestern cluster; and one in the centre/CBD. Washington D.C. (Figure 7c-d) shows a number of complex and geographically overlapping communities. A southeastern cluster (just to the north of the river) sits within the Capitol Hill area; the cluster to the northwest is close to Georgetown and George Washington universities, and may be linked to student movement. These groups appear within both weekend and weekday data. The central cluster covers a range of residential and commercial areas, so it is difficult to speculate on its significance; it may represent commuter behaviours or people travelling to meetings. Again, a time-dependent network analysis might shed some light on this.

### Spatial scaling and spatial community detection

A trend that is strongly apparent in the spatially scaled/residual networks (subfigures a-b of Figures 8-12) is that edges in the city centres are less used that would be expected – the red regions occur in the centre of each of the cities. This could be in part due to a higher density of stands, resulting in the same amount of traffic being spread over a larger number of potential routes; the “gravity” model incorporate codependence on stand activities and hence implicitly on activity density, but it is possible that the correct functional form is not the simple linear one used.

In weekday London (Figure 8a), Waterloo Station predominantly creates flows which terminate in the centre-east of London, known as the City, where the financial services industries are based, and flows perpendicular to that motion (i.e. northwest/southeast) are strongly *under-*represented. This points to the simplicity of the assumptions built into our spatial interaction model; a more accurate model might incorporate office space or job location. However, such a model would also need to account for user demographics or land use at the source and destination stands. Weekday and weekend data (Figure 8b) show that journeys within Hyde Park in the west are over-represented, which would be consistent with tourism and leisure usage. The large flows to and from Waterloo and Kings Cross station that we believe to be commuter-driven flows are notably absent in weekend data. The spatial communities for weekday data (Figure 8c) show a fairly distinct community around Hyde Park and one in the centre of London with smaller communities in the east and south of this group. The eastern community seems to be contained within the financial district, which could point to inter-business transit. Weekend spatial communities (Figure 8d) show a more complex pattern, retaining a group around Hyde Park and identifying new groups in West London.

In Boston (Figure 9a-b), we see flows in the western part of the city that we associate with the region around Boston University (BU); during the week (Figure 9a) we see stronger flows to and from South and North Station than at the weekends (Figure 9b). The spatial community detection picks out the western BU locations in the weekday data (Figure 9c) as well as identifying North and South Station as separate clusters – suggesting their characteristics are unique. This may be due to use of Euclidean distance measures and their unusually high flow volumes – future work could use other community methods, or cosine distance to effectively normalize flows vectors.

Denver’s weekdays (Figure 10a) show a strong overrepresentation from the university area, absent at weekends (Figure 10b). The resulting communities (Figures 10c-d) are correspondingly complex. In weekday Minneapolis (Figure 11a), similarly, the intracampus journey is highlighted; this scaling reinforces the message that this route is much more popular than is explained by a spatial interaction model allocation of cycle journeys, even when weighted for the high stand proximity and stand volumes (in essence, maximum entropy methods spread the flows from a stand evenly across all possible outputs, weighted for destination proximity and destination flow volumes. For these unusually popular *journeys*, they dominate stand popularity and not vice versa; so an even distribution of the source stand’s outputs is not an appropriate analytical approach). It’s likely that students have to travel (e.g. between lectures) more frequently than other sectors of society, overweighting this stand pair. This route is still present at weekends (Figure 11b), but is less dominant; southwestern portions of the city exhibit high volumes. Communities in the weekday data (Figure 11c) tend to cluster around the centre, with the rest of the city being identified with one community. Weekend data (Figure 11d) yields a more complex picture. Washington D.C. (Figure 12a-b) show some small clusters of reciprocal journeys suggesting some hyperlocal usage patterns not identified in this paper; Pentagon City/South Arlington appears again as a predominantly weekday feature (Figure 12a). Weekday data yields a spatial cluster in the centre-west (Figure 12c) and a series of unique stands. Weekend data (Figure 12d) highlights more geographically distinct communities to the centre-east and centre-west.

## Discussion

The analyses of these bicycle sharing system flow networks show important common features and distinctions between the systems. On a trivial level, each system has a different number of stands (nodes), and each city has a distinctive partitioning of its network, which could be related to physical and geographical factors like obstacles such as rivers, weather and climate, and social geographical factors like land use (for examples, the importance of UMN on the pattern of use in Minneapolis) and building density, road type/safety, the general culture of, and attitude to, cycling in each city, and temporal factors such as rush hours, seasons and weekends.

Network analyses typically include betweenness centrality measures – nodes and edges which lie on shortest-paths between nodes. Doing so assumes a relatively low level of connectivity which requires such intermediate nodes. For example, the current London tube network has 306 nodes sharing only 354 edges, resulting in 0.75% of node pairs being directly connected and a mean strength of 2.32. To contrast, bicycle sharing systems are highly connected. London’s 403 stands have nonzero links on 131,475 of the resulting network’s possible 162,409 edges, with a direct connectivity of 80% - other schemes have connectivity in the region of 50–95%. The use of the system does not necessitate “hopping’ between intervening nodes (bar exceptional circumstances) and its embodiment in physical space means that is never faster to do so than pursuing a direct journey; in some senses these systems cannot be considered a “sparse” network and so this measure was not considered.

The simple expedient of using the model fit (in this case, a simple empirical “gravity” model which does not implement e.g. the full richness of a entropy-maximising spatial interaction model – see, for example, [23]) creates a graph of residuals of the fit; this allows flows which appear more strongly than an spatial interaction-type model would predict to become apparent to the researcher or planner. There has been no attempt in this analysis to go beyond the dataset provided and incorporate any land-use data; however, it arguable that such a model would rapidly increase in complexity as we correlate commuters to particular stands with their likely place of work; simply saying e.g. “commuters through Waterloo Station in London are likely to work in the financial district” would provide limited predictive or analytical power above and beyond what has already been demonstrated.

When we compare the systems at a highly aggregate level, we see very similar behaviours based on the distances people tend to travel; system users in Washington and London consistently travel the most similar distance to destinations, in both space and duration. Similarly, there is a consistency in the ratio between the popularity of the top 50 stands in each system. Whether this is specific to this small dataset (5 systems) could be investigated as more data becomes available.

It is may be desirable to further disaggregate these data sets to create time-dependent network analyses which reflect different uses of the scheme at different times of day (as we’ve done for weekdays vs weekends). Work on temporal networks (for example, [24]) often focuses on edges which vary over time *but over which the transmission of the relevant information is instantaneous*. Dealing with both journey times and scheduling (with respect to air travel) was put forward by [25], and may provide a useful model for future analysis. With bikes, the definition of an edge is problematic, and bicycles which undertake long-duration journeys can appear as a persistent edge. A proposed solution to that is to weight the contribution of a journey to each edge as the reciprocal of its duration, and allow the edge to persist throughout the existence of the journey, ensuring that the contribution of that journey to the relevant edge is unity when integrated over all time. This is analogous to the idea of a flow being “[bicycles]/unit time”. These definitional problems are obviated by time-aggregation (i.e. summing over a timescale much larger than that of any individual journey), which is our adopted approach for the analyses in this paper. Similarly, we have considered a very straightforward community detection measure, which may not prove optimal; for example, [26] points to a more sophisticated method which incorporates the concept of overlapping communities in a generalizable way; and in general, there are a host of community detection techniques which go beyond the simple Euclidean-distance out-edge clustering technique we employed.

As commented, a rich seam of exploration is the time-dependence of the network characteristics of these systems, only touched upon in this paper. The possibility for time-dependent identification of changing spatial communities would give a more dynamic picture of the linkages of the city over the course of a day; of course, the danger of disaggregation is the disappearance of meaningful data in the noise of small numbers. Filtering the time period of study for these datasets so that they covered the same month range yielded different patterns of clustering than if all the data from a particular source was used. Identifying tools which balance sensitivity with robustness will be key in consistent analysis, and in using these approaches in planning, strategy or operation.

## Conclusions

We have presented visualisations and analyses of the flows of bicycle sharing system bikes around four North American cities and one European city, demonstrating similarities in the aggregate properties of these systems, and using spatial network techniques to identify local features and communities that exist within these complex spatial networks.

## Supporting Information

File S1 **Supporting Appendices. Appendix 1** Projection and Haversine small-angle approximation. **Appendix 2** Data access and management. **Appendix 3:** Video Visualisations
(DOCX)
