# Supplementary material for: The Structure of Spatial Networks and Communities in Bicycle Sharing Systems
Source: PLoS One. 2013 Sep 6;8(9):e74685. doi: 10.1371/journal.pone.0074685 (PMC3765359; doi:10.1371/journal.pone.0074685)
Supplement: File S1 — Supporting Appendices. Appendix 1 Projection and Haversine small-angle approximation. Appendix 2 Data access and management. Appendix 3: Video Visualisations (DOCX) [file pone.0074685.s001.docx]

**Appendix 1: Projection and Haversine small-angle approximation**

All geographical visualisations presented in this paper, use an equirectangular projection (x = aλ, y = bφ;), and the ratio a:b is set to Δλcos(Δφ):Δφ. Over the scale of the cities involved, this creates limited distortion.

We based our journey calculations upon the Euclidean displacement, and as such are not direct analogues of journey distance. To calculate these displacements, we employed the Haversine formula, using small-angle approximations (sin x = x and cos(x)cos(y) = cos^2^(0.5(x+y))) and assume a constant radius of 6378km. This introduces errors less than 1 part in 10^4^, or 2m in 20km; variations in the radius of the earth could account for variations >10m on the same distance scale, and routing would have a much larger impact on actual distance travelled.

**Appendix 2: Data access and management**

Data was stored in a MySQL database; each city had a series of tables (illustrated in Table 3). This configuration allowed for rapid slicing of data by time of day, weekday/weekend, etc and allowed interfacing using Sequel Pro for numerical prototyping and generating CSV outputs, MATLAB for analysis, modelling and graphing, and Processing for visualization, the latter two through standard JDBC connector classes.

London’s data required special handling. The journey data and stand locations for London were obtained from the city’s transport authority, Transport for London (TfL), as a series of data files. A custom set of IDs were created for the stands, allowing certain groups of stands, which are operationally separate but physically adjacent, to be combined together and treated as a single stand for this analysis. For example, the Waterloo “superdock”, which has 126 stands, has three IDs in TfL’s data, each corresponding to around 40 of the stand’s docking points. Such adjacent stands are generally identified by having identical names but with different numeric suffices, for example Waterloo Station 1, Waterloo Station 2.

Journeys with likely faulty data were also removed – this includes journeys apparently finishing before they started, very short journeys which finish at the same stand (indicating a detected fault) and journeys over 24 hours, which is the maximum allowed journey time for users of the system (most journeys are much shorter; for London, 90% of journeys are less than 25 minutes long, 98% of journeys are less than an hour, and 99.5% of journeys are less than two hours). Journeys that may refer to operator redistributions, identified in the data by bicycles having an ID of 0, were also removed from the data, and set aside for later analysis. London was also the only city data for which time was stored in UTC – so it was very important to set all data to local time (i.e. incorporating the shift to and from British Summer Time). For other city data, we have less detailed local knowledge, and so basic data cleaning was performed; data appeared in some variant of local time, simplifying data cleaning.

With all of these data sources, some distortion is introduced by the phased introduction of the system’s stands; some stands existed at the end of the reporting but were not present at the beginning. It is possible to normalise newer locations based on the proportion of time they’ve existed for, but this would result in a state that may not be representative of the real mechanism of uptake and builds in additional assumptions. This phased introduction of stands will likely create some impact on the detailed network of flows, but we can anticipate it having a limited effect on the network statistics. It is important to note that we did not include data from London collected after the “Phase 2” expansion into east London and Canary Wharf in March 2012, which would have represented a major change.

**Appendix 3: Video Visualisations**

These visualisations were all prepared with real journey data using Processing to call data from MySql database tables, using Routino to generate inferred routes as described in the text of the paper.

S1. Zaltz Austwick M, O’Brien O (2011) Last Xmas I gave you my... bike? Available: http://vimeo.com/33775737. Accessed 22 November 2012.

Visualisation of travel on Xmas Day 2010, based on London Bike Share data and Routino inferred routes.

S2. Zaltz Austwick M, O’Brien O (2012) Boston Bikeshare Sunday October 9th 2011. Available: http://vimeo.com/47530681. Accessed 22 November 2012.

Visualisation of Boston Bike Share Data on October 9^th^ 2011.

S3. Zaltz Austwick M, O’Brien O (2012) Minneapolis Bike Share - October 9th 2011. Available: http://vimeo.com/47531275. Accessed 22 November 2012.

Visualisation of Minneapolis Bike Share Data on October 9^th^ 2011, chosen to coincide with the Boston Bike Share visualization above.

S4. Zaltz Austwick M, O’Brien O (2012) All Weekday London Bikeshare Data on Vimeo. Available: https://vimeo.com/54079850. Accessed 12 February 2013.

This shows all bikeshare data for the November 2010-May 2011 period shown on one day, but with correct times. This generates an aggregate picture of scheme activity for a summed (or equivalently, averaged) day.
